# Flies as Disease-Carriers

**Published:** 1908-12-19

**Authors:** 


### The Hospital

A Journal of

The Medical Sciences and Hospital Administration.

New Series. No. 93, Vol. IV. [No. 1165, Vol. XLV.] SATURDAY, DECEMBER 19, 1908.

### FLIES AS DISEASE-CARRIERS.

It is not unnatural, after what has been demonstrated in connection with malaria, yellow fever, and trypanosomiasis, that an attempt should be made to fasten upon the common flies of this country responsibility for the dissemination of various infective maladies. There is no doubt that these flies are admirably adapted for the fell purpose of distributing pathogenic organisms, for they are ubiquitous, daring, and catholic in their appetites. Moreover, the simple experiment of allowing a fly to crawl over the surface of an agar plate will convince anyone that the fly habitually harbours organisms capable of developing at body heat. When, therefore, it is shown that a disease like the summer diarrhœa of infants is synchronous with the height of the fly season, there is some excuse for a rapid leap to the conclusion that flies are the agents by which the infection is disseminated.

The opening for error in this somewhat hasty deduction is obvious, but not of so much moment as may at first sight appear. Even though flies be really innocent of conveying the specific organism—if there is but one—which excites summer diarrhœa, it is amply demonstrated that foodstuffs to which they have had access tend to putrefy far more quickly than similar materials kept under similar conditions but protected. In a recent issue of the *Lancet*, Dr. Nash, County Medical Officer of Health for Norfolk, published an account of some simple experiments designed to demonstrate this fact. On a warm day in August two clean saucers were filled with milk from the ordinary household supply. They were placed side by side on a table in the kitchen, one being covered, the other left exposed to the air. In all other respects the conditions in the two cases were identical. Five hours later the experiment terminated, when the uncovered saucer was found to contain the bodies of two flies. The two milks were then plated out on gelatin and incubated for 42 hours at 22° C. The results of this procedure led to the estimate that the uncovered milk contained rather more than twice as many organisms as its covered neighbour. A further point of importance lay in the subsequent behaviour of the two samples. Within 24 hours the uncovered specimen was slightly putrefactive in odour, and was stinking in 48 hours; while the covered specimen was odourless at the expiry of 24 hours, and in 48 hours had no smell except that of sour milk. It does not, of course, follow that the putrefactive organisms which gained access to the uncovered specimen were brought there by the flies found drowned in it, for they may have been deposited from the air; but the experiment does decidedly bring flies under grave suspicion. At all events, whether flies carry the contagion of summer diarrhœa or not, the *primâ-facie* case against them on other grounds is sufficiently strong to warrant a campaign against them. This campaign has already begun in many parts of the country. Last year Dr. Hamer, one of the medical officers of the London County Council, presented a report on the extent to which the fly nuisance was produced by accumulations of offensive matter. A further report from the same source lies before us, and gives some interesting information. We may note that Sir Shirley Murphy, in his introduction, enters a caveat against the final acceptance, on present evidence, of the view that the fly acts as carrier of the diarrhœa organism. The substance of the supplementary report is briefly this. More than nine-tenths of the flies caught were examples of the common house fly (*Musca domestica*), but the smaller house fly (*Homalomyia canicularis*) and a biting fly (*Stomoxys calcitrans*) were also found in considerable abundance. Of the centres of observation four were in the neighbourhood of premises in which stable manure or house refuse, or both, were manipulated. These centres yielded a far greater number of flies than the other areas investigated. Dr. Hamer observes that the part played by stables in determining fly prevalence is one of paramount importance, and suggests the propriety of instituting formal control over them from this point of view. Nevertheless it does not appear that deaths from diarrhœa occur with greater frequency among populations living in the near neighbourhod of stables than among those elsewhere. In a population of 5,000, living in a part of Finsbury surrounding large stables (which formed one of the centres of observation for this report), there occurred during the summer of 1908 only four deaths from diarrhœa; and similar figures are reported from Islington and Southwark. Further, although in 1908 the mortality from summer diarrhœa was more than twice that of 1907 (an unusually cool summer), the number of flies was not correspondingly excessive.

It is pretty clear, therefore, that we have not yet established the specific guilt of the domestic fly. His potentialities for evil are no doubt limited by many considerations, some of them largely accidental, such as the amount of infected material existent, and the ease of access both to it and to the articles of food, particularly sugar and milk, by which it is conveyed to fresh victims. But enough has been found against the fly to justify his condemnation not only as a nuisance, but as a danger. The problem of his elimination, if success is to be hoped for, must be attacked at its root—that is to say, the assault must fall upon the breeding-places of flies, upon the accumulations of organic filth, like manure and household refuse, which experiment has shown to be such efficient hatcheries for these pests. In the meanwhile we must continue to urge upon the poorer mothers in our cities the harm which may befall from the practice of keeping milk uncovered upon a shelf, and of allowing flies to crawl upon the sugar intended for infant consumption.

